# Group-based trajectory modelling of multiple health outcomes in a cost–consequence framework: a randomized controlled trial of a remote person-centred care intervention for people with common mental disorders in Sweden

**DOI:** 10.1186/s41687-026-01043-y

**Published:** 2026-03-20

**Authors:** Benjamin P. Harvey, Tayue T. Kebede, Andreas Fors, Matilda Cederberg, Sara Alsén, Emmelie Barenfeld, Marlinde Lianne van Dijk, Hanna Gyllensten

**Affiliations:** 1 Institute of Health and Care Sciences, Sahlgrenska Academy, University of Gothenburg, Gothenburg, Sweden; 2 University of Gothenburg Centre for Person-Centred Care (GPCC), Sahlgrenska Academy, University of Gothenburg, Gothenburg, Sweden; 3 School of Public Health and Community Medicine, Institute of Medicine, Sahlgrenska Academy, University of Gothenburg, Gothenburg, Sweden; 4 Region Västra Götaland, Research, Education, Development and Innovation, Primary Health Care, Gothenburg, Sweden; 5 Department of Psychotic Disorders, Sahlgrenska University Hospital, Gothenburg, Sweden; 6 Institute of Neuroscience and Physiology, Department of Health and Rehabilitation, Sahlgrenska Academy, University of Gothenburg, Gothenburg, Sweden; 7 Department of Public and Occupational Health, Amsterdam UMC, Location Vrije Universiteit Amsterdam, Amsterdam, Netherlands; 8 Amsterdam Public Health research institute, Health Behaviours & Chronic Diseases, Amsterdam, Netherlands

**Keywords:** Telemedicine, Patient-Centered Care, Person-Centred Care, Person-Centered Care, Costs and Cost Analysis, Mental Health, Group-Based Trajectory Modelling, Common Mental Disorders, eHealth

## Abstract

**Background:**

Evaluations of complex interventions are increasingly used within the health and social sciences and form an integral part of the ongoing research into person-centred care (PCC). The longitudinal nature of these interventions, measuring multiple outcomes over time, is often challenged by traditional economic frameworks when informing decision-makers. Therefore, the aim of this study is to explore how group-based trajectory modelling (GBTM) can be used to evaluate *multiple* outcomes within economic evaluations, specifically when outcomes are measured longitudinally and lack an established summary metric.

**Methods:**

GBTM and a cost-consequence analysis (CCA) were performed on two-year data from the PROMISE randomised controlled trial, a remote PCC add-on intervention combining a web-based platform and telephone support for people on sick leave due to common mental disorders. Group trajectories were modelled combining General Self-Efficacy (GSE), EQ-5D values, the Perceived Stress Scale (PSS), and the Shirom-Melamed Burnout Questionnaire (SMBQ). Costs were reported from a societal perspective for trajectory groups, and within a CCA summarising healthcare use, prescription drugs, productivity loss, and quality-adjusted life years (QALY).

**Results:**

The intervention group had lower total and mean costs for primary care, prescription drugs, and productivity loss compared to the control group at two years. GBTM identified four groups: *High*,* Moderate*,* Low*, and *No Improvement*. The *High Improvement* group, of whom 53% belonged to the intervention group, had the greatest improvements across all outcome measures and the lowest mean costs. The *No Improvement* group experienced the worst baseline health as well as significant differences in education level and primary diagnosis. No statistically significant differences were found between the intervention and control groups in relation to trajectory group allocation. GBTM and QALY differences were sensitive to imputation and value sets.

**Conclusion:**

This study demonstrates that GBTM coupled with CCA, offers a practical framework when evaluating complex interventions where multiple outcomes evolve over time. Understanding the importance of sociodemographic factors and heterogeneous response patterns meaningfully enriches economic evaluation and can help guide the development and evaluation of person-centred, complex interventions.

**Clinical Trial Number:**

NCT03404583 - ClinicalTrials.gov.

**Supplementary Information:**

The online version contains supplementary material available at 10.1186/s41687-026-01043-y.

## Background

Complex interventions are commonly used within health and social care sciences to facilitate a better understanding of the impact of an intervention across multiple levels of influence, from individual to societal [1]. What constitutes a complex intervention is determined by several properties: the number of components, the range of actions or practices, the expertise and skills, the groups, settings, or levels targeted. This overarching approach to investigation forms an integral part of the ongoing research within person-centred care (PCC), with the goal of influencing both individual and organisational change for those who receive and provide care within different settings [2, 3]. However, complexity of an intervention can create complexity in effective evaluation, and further knowledge is needed in order to present research findings that capture the broad impact of these interventions.

Health economic evaluation of PCC interventions are an evolving field, faced with several challenges due to the multiple components that act either independently, or in conjunction with one another [4]. Standard economic evaluations, such as cost-effectiveness or cost-utility analysis, treat patients as a homogeneous group, reporting costs and effects of alternatives as average point estimates for each group with uncertainty. Subgroup analysis can alleviate some of these problems by addressing heterogeneous groups within the main sample, however, these are often based on pre-determined characteristics, such as age and sex, that are not influenced by the intervention [1]. The effective evaluation of complex interventions, that are longitudinal in nature, provides a significant opportunity to understand the meaningful subgroups within a study population that arise from distinct developmental trajectories, which are not identifiable ex ante based on individual characteristics [5]. Trajectory analysis, evaluating multiple outcomes, has not been adopted within a cost-consequence framework, but could provide valuable insights when informing decision-making and resource allocation.

Commonly, researchers are faced with the dilemma of how to best explore and communicate the richness of measurements at their disposal in a cohesive manner. Group-based trajectory modelling (GBTM) is a statistical method that maps the evolution of developmental trajectories as they occur over time, allowing researchers to classify groups based on their differences when responding to an intervention and allowing for the inclusion of multiple outcome measures simultaneously [5]. Research into core outcomes within PCC [6] has demonstrated that stakeholders value a wide range of outcome measures that evaluate the effect of healthcare beyond the direct patient, healthcare professional (HCP) interaction. This further emphasises the need for economic evaluations to incorporate multiple outcomes rather than relying on a single metric. Additionally, GBTM can add an informative layer to economic evaluations by presenting costs together with developmental trajectories. This approach has been explored in a study by Blanck et al. [7], which used GBTM for both costs and general self-efficacy (GSE) in the evaluation of a PCC intervention for patients with chronic obstructive pulmonary disease and chronic heart failure, showing that participants with improving or stable GSE trajectories were associated with lower costs.

Building on these methodological insights, this study will model patient trajectories incorporating multiple outcomes including GSE, EQ-5D values, the Perceived Stress Scale (PSS), and the Shirom-Melamed Burnout Questionnaire (SMBQ), alongside costs, with data from the PROMISE study, a remote PCC add-on intervention combining a web-based platform and telephone support for people on sick leave due to common mental disorders (CMD). Previous research [8] conducted on this randomised controlled trial reported a significant difference in both the intention-to-treat and per-protocol groups at three months based on the composite endpoint of changes in GSE and level of sick leave. Additionally, a cost-utility analysis using costs and effects at one year reported the intervention to be cost-effective i.e. dominant (the intervention was less costly and more effective than its comparator) [9].

## Methods

### Aim

The aim of this study is to explore how GBTM can be used to evaluate *multiple* outcomes within economic evaluations, specifically when outcomes are measured longitudinally and lack an established summary metric.

### Design

GBTM and a cost-consequence analysis (CCA) were performed using data from the PROMISE randomised controlled trial (ClinicalTrials.gov-registration: NCT03404583), a remote PCC add-on intervention combining a web-based platform and telephone support for people on sick leave due to CMD [10]. Healthcare and societal costs, as well as outcome data were analysed over a two-year time horizon. More in-depth information on the study methods and procedures, as well as the main trial results based on the primary outcome measure (a composite score of changes in GSE and sick leave), have been published elsewhere [8, 10]. The reporting was performed in accordance with the Consolidated Health Economic Evaluation Reporting Standards criteria [11] and the Consolidated Standards of Reporting Trials guidelines [12].

### Study setting, participants and procedures

The study included nine public primary healthcare centres in a socioeconomically diverse area of western Sweden, from which patients on sick leave due to CMD were recruited. Participants were considered eligible for the study if they were aged 18–65 and were currently on sick leave due to a physician-diagnosed CMD as defined by the International Statistical Classification of Diseases (ICD) and Related Health Problems, Tenth Revision (mild to moderate depression (F32 and F33), mild to moderate anxiety disorder (F41), reaction to severe stress, and adjustment disorders (F43, except posttraumatic stress disorder), which includes the Swedish diagnosis exhaustion disorder (F43.8 A). These disease classifications were selected due to their high prevalence amongst patients on sick leave due to CMD. Further inclusion criteria were; the current sick-leave episode must not have exceeded 30 days; patients had to be employed or studying at least part-time during the previous nine months; and they had to have a registered address in Sweden and be able to manage the Swedish language. The exclusion criteria were: (1) previous sick leave due to depression, anxiety disorders and stress reactions and disorders exceeding 14 days over the previous three months; (2) severe impairment that hindered the use of a digital device, preventing participation in the eHealth support; (3) expected survival of < 12 months; (4) ongoing, documented diagnosis of alcohol or drug abuse; (5) diseases that could potentially interfere with the planned follow-ups; (6) assessment of the intervention as a burden; and (7) participation in any conflicting studies.

To achieve 80% power with an alpha error of 0.05, a sample size of 91 patients in each group was calculated to achieve a 20% to 40% improvement in the primary composite endpoint, in the control and intervention groups, respectively [8, 10]. In order to account for potential attrition, 110 participants were recruited to each study group.

Recruitment of the participants took place between February 2018 and June 2020 [8]. After screening of medical records from the aforementioned nine participating healthcare centres, a total of 1317 people were assessed for eligibility with 315 not meeting the inclusion criteria. Eligible participants (*n* = 1002) were posted a letter containing information about the study and could initiate first contact following the instructions on the information letter or were contacted via phone by a HCP. A further 787 either did not respond (*n* = 199) or declined to participate in the study (*n* = 588) (Supplementary Fig. 1). A final group of 215 people accepted to participate and were sent an informed consent form and information about their rights as participants by standard post. Randomisation was conducted applying computer-generated random numbers, had a 1:1 allocation ratio, and was stratified by age (< 50 or ≥ 50), and diagnostic group (depression, anxiety disorders, stress reactions and disorders). The intervention group consisted of 107 participants with 108 being allocated to the control group. Six participants withdrew after the study commenced, leaving 102 in the intervention group and 107 in the control group for analysis. Further details of the recruitment process have been published previously [10].

### Remote PCC add-on intervention and usual care

Both the intervention and control groups received usual care through local primary and specialised care centres in accordance with current medical guidelines. In addition, the intervention group was provided with a remote PCC add-on intervention for six months comprising a digital platform and structured telephone support. The digital platform, developed by the University of Gothenburg Centre for PCC, was designed to operationalise PCC in line with the Gothenburg Model [13]. The intervention commenced with the co-creation of a personalised health plan through a telephone conversation with an HCP dedicated to providing the intervention. The health plan, grounded in the patient’s narrative, outlined individual goals, resources, and needs, as well as plan between the HCP and the patient to collaboratively achieve set goals. Structured telephone support included follow-up calls comprised of discussions focused on supporting the patient in their recovery process and achieving health goals. Follow-up meetings via telephone were scheduled based on the patient’s preferences and provided opportunities to revise and modify the health plan when applicable in accordance with any changes to their needs or goals. The digital platform facilitated two-way communication between the patient and HCP, utilising private messages, whilst providing access to the documented health plan, self-rated health scores, and other healthcare resources (e.g. links to disease-specific information, support centres). Both the patient and the HCP had access to the digital platform for the entirety of the six month intervention period (access to the digital platform was still available after six months, although the telephone support had ceased), with the patient invited to customise access that allowed for informal carers, family, or friends to view the information. The key distinctions between groups were that control participants did not co-create a health plan, receive the structure PCC telephone support, or have access to the digital platform. A more detailed description of the intervention has been published elsewhere [8, 10].

### Data collection

Postal questionnaires were used to collect self-reported sociodemographic data at baseline, and outcome measures, including the GSE, EQ-5D (the three levels of response version i.e. EQ-5D-3 L), PSS and the SMBQ at baseline, three, six, 12, 18 and 24 months after randomisation. Patient-level data were collected from several patient registries; (1) socio-economic variables were obtained from the Longitudinal Integrated Database for Health Insurance and Labour Market Studies, held by Statistics Sweden; (2) utilisation of primary care, specialised outpatient care, inpatient care, including diagnosis-related groups (DRG) from the VEGA register, held by Region Västra Götaland; (3) amounts and costs of dispensed pharmaceuticals from the Swedish Prescribed Drug Register, held by the National Board of Health and Welfare (NBHW); (4) cause and time of death from the Cause of Death Register, NBHW; and (5) compensation for absenteeism due to disability and/or illness from the Micro Data for Analysis of the Social Insurance System (MIDAS) register, held by the Social Insurance Agency. Data extracted from the digital platform and project-specific communications list for telephone calls were used to estimate the time allocated by HCP.

### Costs

Adopting a societal perspective over a two-year time horizon, included costs were productivity loss, inpatient, outpatient and primary care, prescription drugs, and the costs associated with the intervention. Unit costs for primary care were calculated using the weighted cost per healthcare contact as recommended by the Swedish Association of Local Authorities and Regions’ Statistics for Healthcare and Regional Development, 2022 (Supplementary Table 1) [14]. Per-patient total healthcare costs for inpatient care and specialised outpatient care were calculated by multiplying the 2018–2021 DRG weights, by the average cost per healthcare visit in 2022 [15]. For psychiatric encounters, psychiatric care-specific DRGs were used, whereas for other encounters, somatic care-specific DRGs were applied. Psychiatric care encounters included those where the HCP was a medical social worker, specialised psychiatrists, psychologists, or where the main diagnosis belonged to the ICD chapter F. Encounters in specialised outpatient care with missing DRG weights were imputed using the median cost of encounters of the same type (HCP/care level) and whether they were assessed as psychiatric or somatic encounters (Supplementary Table 2). Given the skewed distribution observed in healthcare costs, the median was considered a more robust summary measure than the mean. Imputation for inpatient care costs was based on the median per-day cost. Amounts and costs of dispensed pharmaceuticals were calculated using the total cost including patient fees. Applying the human capital approach, productivity loss from sick leave was estimated by multiplying the reported absenteeism from work, by the mean wage based on 10-year age categories and social security contribution [16, 17]. The intervention costs were calculated based on the mean monthly salary of a research nurse (SEK 38 400) including social contributions (46.45%). All second-year costs were discounted by 3%.

### Outcome measures

Cronbach’s alpha was used to assess the internal consistency of the self-reported outcome measures GSE, PSS and SMBQ (Supplementary Table 3).

#### General self-efficacy (GSE)

GSE is a 4-point scale with 10 items (total score ranging from 10 to 40) that measure situation-specific beliefs and participants’ competence in responding to and controlling environmental demands and challenges [18]. Participants are considered to have improved their GSE with an increase of ≥ 5 points. GSE is regarded as unchanged if the score is less than five points in either direction, whereas a deterioration in GSE is a decrease of ≤5 points. This corresponds with previous research [19, 20] that suggests five points as a threshold for minimal important change based on reported standard deviations in general population studies. Internal consistency of the GSE was assessed using Cronbach’s alpha and was excellent (α = 0.91).

#### EQ-5D

The EQ-5D instrument serves as a tool for summarising and comparing EQ-5D values, facilitating the calculation of quality-adjusted life years (QALYs) [21, 22]. The EQ-5D has five dimensions: mobility, self-care, usual activities, pain/discomfort, and anxiety/depression. Within each dimension, the respondent can choose from one of three health states; (1) no problems, (2) some problems, or (3) severe problems [22]. Final scores were converted to EQ-5D values using the UK preference-based and the Swedish experience-based value sets [23, 24], to reflect both current policy practice and methodological recommendations. QALYs were calculated over the two-year study period using the area-under-the-curve approach with second year effects discounted at 3%.

#### Perceived stress scale (PSS)

The PSS is widely considered the gold standard instrument for measuring stress perception [25]. This self-reported questionnaire is used to assess the degree to which a respondent finds circumstances in their life affecting feelings and thoughts for the past month, indicating that a relatively recent time frame is assessed [26]. This study applied the 14-item PSS which scores each item on a 5-point Likert scale from 0 = Never to 4 = Very Often. Seven questions on the PSS use reverse scoring (four, five, six, seven, nine, ten, and 13). Total scores can range from 0 to 56, with low scores indicating low stress (Low Stress (scores 0–18), Moderate Stress (scores 19–37), High Stress (scores 38–56)). The Cronbach’s alpha assessment for internal consistency was acceptable (α = 0.78).

#### Shirom melamed burnout questionnaire (SMBQ)

Burnout is a long-term affective state consisting of emotional exhaustion, physical fatigue, and cognitive weariness resulting from prolonged exposure to stress, particularly work-related stress [27]. Burnout was assessed using the SMBQ where respondents are asked to report based on four subscales, physical fatigue (8 items), cognitive weariness (6 items), tension (4 items) and listlessness (4 items), on a 7-point scale (1 = almost never to 7 = almost always). Five items on the scale use reverse scoring (1 = Tension, 3 = Listlessness, 1 = Physical Fatigue). However, according to Lundgren-Nilsson et al. [27], removing the subscale *Tension* and its four items, thereby having an 18-item scale, has higher internal consistency reliability estimates. For each sub-domain, and the scale as a whole, the score is averaged by dividing by the number of items. An average score of 4.4 on the scale is suggested as a suitable guide in determining clinical burnout [27]. Assessing internal consistency using Cronbach’s alpha was excellent (α = 0.95).

### Imputation

At any measurement point, a single missing response to the questionnaires resulted in the inability to calculate total average scores across all measurement points. Missing questionnaire values at baseline were imputed using mean imputation independent of treatment allocation. Mean imputation has been recommended as more efficient than multiple imputation at baseline as it ensures that imputed values are equally distributed among the population and independent of treatment allocation [28]. After stratifying by treatment group, single imputations were calculated and applied to the GBTM, whereas multiple imputations were generated for comparison and in calculating QALYs (Supplementary Table 4). Predictive mean matching with the Multivariate Imputation by Chained Equations R package was used to perform the imputations [29].

### Analysis

#### Group-based trajectory modelling (GBTM)

GBTM is a statistical method that aims to identify a finite number of groups of individuals following similar trajectories over time for either single or multiple outcomes [5]. The first step was to estimate the number of trajectory groups for GSE, EQ-5D values, PSS, and SMBQ separately. In order to assess the correct number of trajectory groups, each outcome was tested beginning with two groups, with additional groups being added to assess model fit. This was tested alongside linear, quadratic, or cubic trajectory specifications. Following the recommendations of Nagin et al. [5] and Wheeler [30], an average posterior probability (APP) of ≥ 0.7 for all groups guided the diagnostic process of model selection. Although tested separately, the objective is not to identify the preferred model for each outcome individually, but instead to clarify the types of distinctive trajectories that should be represented in the multi-trajectory model. After testing each outcome individually, the four outcomes were modelled together, informed by the individual tests and APP criteria. Trajectory analyses were performed using Stata SE version 19.5 [31].

#### Statistical analysis of trajectory groups

Continuous variables were checked for homogeneity of variance using Levene’s test and analysed using a standard one-way ANOVA. Post-hoc pairwise comparisons were conducted using Tukey’s test to identify specific group differences. Categorical and binary variables were analysed using chi-squared or Fisher’s exact tests as appropriate. For categorical variables with significant global tests, pairwise Fisher’s exact tests were conducted between all trajectory group combinations with *p*-values adjusted using the Holm method to control for multiple comparisons. Proportional differences between intervention and control groups within each trajectory were examined using two-proportion z-tests. Household disposable income was adjusted for inflation to 2022 prices [32]. QALY values under multiple imputation were evaluated using pooled linear models. Statistical analyses were performed using R version 4.2.2 for statistical analysis [33].

#### Sensitivity analysis

The sensitivity of group allocation was explored using EQ-5D values from the Swedish experience-based value set when predicting trajectory groups. The same stepwise process for GBTM was performed as described previously.

#### Cost-consequence analysis (CCA)

A CCA is a health economic framework that presents costs and consequences of alternative interventions in a disaggregated manner. This structure allows investigators to examine how specific costs relate to individual outcomes and process measures, thereby supporting a more nuanced assessment of an intervention’s overall impact across multiple dimensions [34]. The CCA in this study reported the distribution between the control and intervention arms of the study, separated by trajectory groups, costs, and QALYs.

## Results

Demographic characteristics showed no statistically significant differences between the control and intervention groups at baseline (Table 1). Mean costs at two years were lower for the intervention group in primary care, SEK 48 765 (CI 41 197, 56 154) compared with the control group, SEK 52 129 (CI 45 039, 59 218), prescription drugs, SEK 8 324 (CI 4 154, 12 494) in the intervention group and SEK 43 405 (CI -19 684, 106 494) in the control group, and productivity loss, SEK 202 370 (CI 153 192, 251 548) and SEK 213 081 (CI 164 188, 261 973) in the intervention and control, respectively (Table 2).

**Table 1:** Demographic Characteristics

Characteristics	Control group (*n* = 107)	Intervention Group (*n* = 102)
Age (years), mean (SD^1^)	42 (12)	42 (11)
Female, n (%)	93 (87)*	82 (80)
**Civil status**,** n (%)**		
Living alone	30 (28)	40 (39)
Married or living with partner	77 (72)	62 (61)
**Education level**, n (%)*		
Compulsory	7 (7)	6 (6)
Secondary school	16 (15)	21 (21)
Vocational college	20 (19)	15 (15)
University	63 (59)	59 (58)
**Country of birth**,** n (%)**		
Sweden	91 (85)	89 (87)
Other	16 (15)	13 (13)
**Diagnosis (ICD**^**2**^**)**,** n (%)**		
Stress (F43)	69 (64)	65 (64)
Depression (F32 & F33)	23 (21)	21 (21)
Anxiety (F41)	15 (14)	16 (16)
**Disposable Income**,** mean (SD)**		
Household^3^ (SEK^4^)	356 976 (243 200)	344 690 (112 919)

^1^SD: standard deviation, ^2^ICD: Internation Classification of Functioning, ^3^Household: Disposable income per unit of consumption of a household, inclusive income earnt in another Nordic country, ^4^SEK: Swedish Crowns, ^*^Missing value

**Table 2:** Total and mean costs at two years

	Control Group		Intervention Group	
Care Type	Total Cost (SEK^1^)	Mean (CI^2^)	Total Cost (SEK)	Mean (CI)
Primary Care	5 577 751	52 129 (45 039, 59 218)	4 964 879	48 675 (41 197, 56 154)
Specialised Outpatient Care	3 834 985	35 841 (24 643, 47 039)	4 439 813	43 528 (24 463, 62 592)
Prescription Drugs	4 644 359	43 405 (-19 684, 106 494)	849 030	8 324 (4 154, 12 494)
Productivity Loss	22 799 612	213 081 (164 188, 261 973)	20 641 687	202 370 (153 192, 251 548)
Intervention Costs*			88 831	871 (765, 977)
Total Costs	36 856 707		30 984 240	

^1^SEK: Swedish Crown, ^2^CI: Confidence Interval *including unplanned intervention costs

### Trajectory groups

**Fig. 1:**
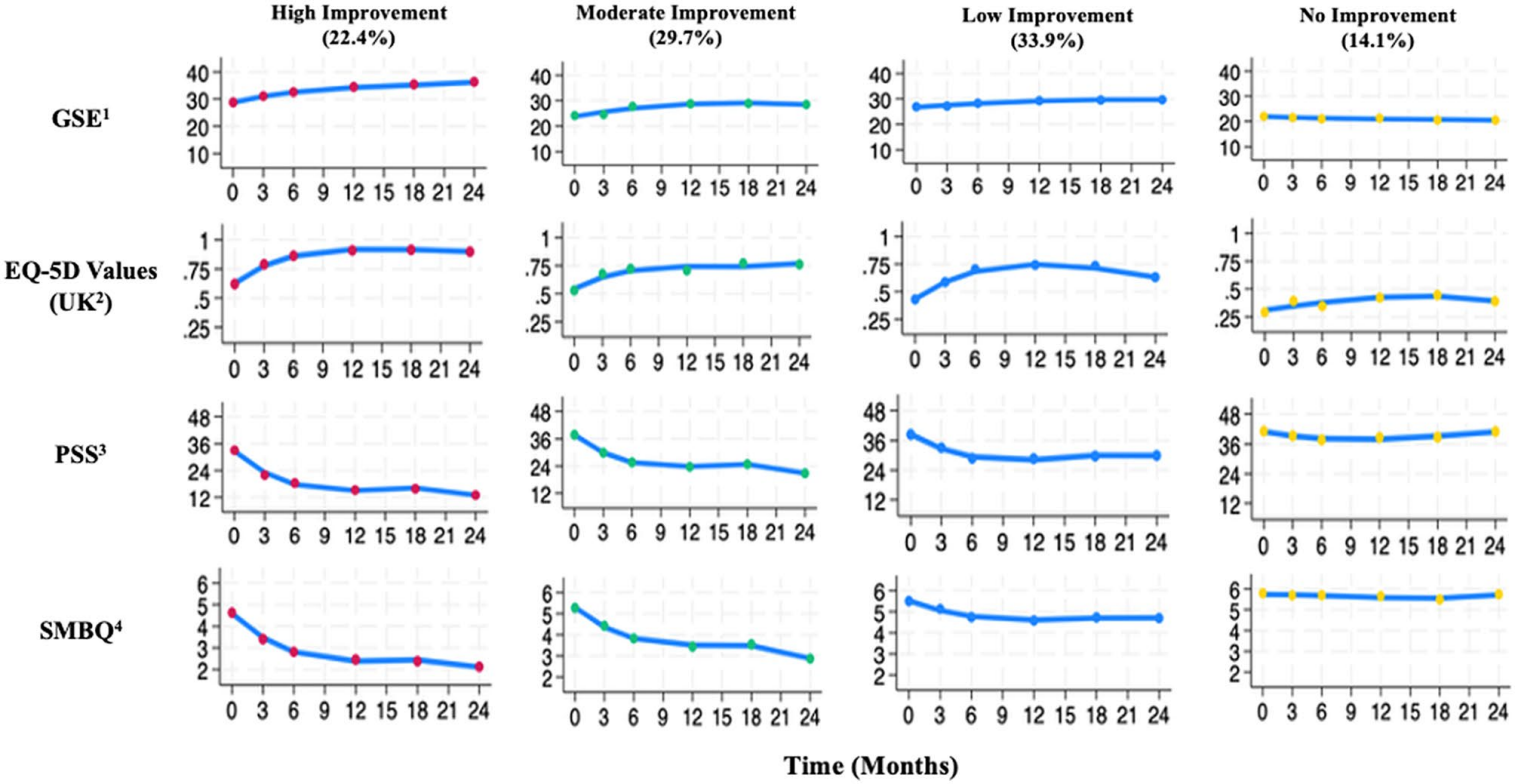
Model-estimated means for group-based trajectories over two years. ^1^GSE: General Self-Efficacy, ^2^ UK: United Kingdom, ^3^ PSS: Perceived Stress Scale, ^4^ SMBQ: Shirom-Melamed Burnout Questionnaire

GBTM resulted in four trajectory groups; *High Improvement Group*, *Moderate Improvement Group*,* Low Improvement Group* and the *No Improvement Group* (Fig. 1). Group selection was specified as a cubic (third-order) function with a high classification quality based on APP scores > 0.9 across all groups (Supplementary Table 5). The observed model fit for the data also scored high, with the observed outcome and predictor outcomes aligning for all groups. A more detailed description of the process, including reference to the source code, can be found in the supplementary material.

The *High Improvement Group* (*n* = 47) was defined by having the greatest overall improvements in all outcome measures, including transitioning from moderate to low stress in the PSS, and moving below the 4.4 clinical burnout threshold according the SMBQ. The *Moderate Improvement Group* (*n* = 64) presented with similarly positive results compared with the *High Improvement Group*, however, resulted in lower overall changes in GSE and EQ-5D values at two years. Additionally, the *Moderate Improvement Group* moved outside the clinical burnout range, although remained within the moderate stress range throughout the study based on the PSS. The *Low Improvement Group* (*n* = 69) is characterised by slight improvements in GSE, EQ-5D values, PSS, and SMBQ over the first 12 months that then remained stable or regressed. The *Low Improvement Group* members remained in the moderate stress PSS category as well as not falling below the 4.4 range for clinical burnout throughout the study. The final trajectory group, the *No Improvement Group (n = 29)*, despite small increases in EQ-5D values and PSS during the initial 12-months, had outcome scores that reverted back to baseline measures or were unchanged. The *No Improvement Group* is also characterised by high stress on the PSS and clinical burnout according to the SMBQ. Post-hoc statistical differences can be found in the supplementary material (Supplementary Table 6).

### Trajectory group demographics

Baseline demographics showed significant differences in education level across trajectory groups (*p* = 0.03) (Table 3). Participants in the *High Improvement Group* were more likely to hold a university degree (72%) compared with those in the *No Improvement Group* (21%). Diagnosis was also statistically significant (*p* = 0.05) with stress-related disorders (F43) being the most common primary diagnosis across all groups; however, depressive disorders (F32–F33) were more frequent among participants in the *No Improvement Group* (38%) compared with the other trajectory groups (12–22%). The *No Improvement group* had the highest percentage of patients living alone (48%), whilst the *High Improvement Group* had the largest disposable income. Statistically significant differences were also observed in all baseline outcome measures (*p* < 0.01). Participants in the *High Improvement Group* reported higher GSE and EQ-5D values, along with lower SMBQ and PSS scores. Conversely, participants in the *No Improvement Group* exhibited the poorest baseline outcome measures across all domains.

**Table 3:** Trajectory Group Demographics

Characteristics	No Improvement (*n* = 29)	Low Improvement (*n* = 69)	Moderate Improvement (*n* = 64)	High Improvement (*n* = 47)	*p* value
Intervention Group (%)	14 (48)	32 (46)	31 (48)	25 (53)	0.9
Age (years), mean (SD^1^)	42.8 (11)	41.7 (11.6)	42.1 (11.9)	42.9 (11.2)	0.9
Female, n (%)	24 (83)	60 (87)	53 (83)	38 (81)	0.8
**Civil status**,** n (%)**					
Living alone	14 (48)	18 (26)	24 (38)	14 (30)	0.15
Married or living with partner	15 (52)	51 (74)	40 (62)	33 (70)	
**Education level**, n (%) *					
Compulsory	2 (7)	1 (1)	9 (14)	1 (2)	0.03
Secondary school	5 (17)	11 (16)	13 (20)	8 (17)	
Vocational college	16 (55)	18 (26)	7 (11)	4 (9)	
University	6 (21)	38 (55)	34 (53)	34 (72)	
**Country of birth**,** n (%)**					
Sweden	25 (86)	59 (86)	55 (86)	41 (87)	0.99
Other	4 (14)	10 (14)	9 (14)	6 (13)	
**Diagnosis (ICD**^**2**^**)**,** n (%)**					
Stress (F43)	14 (48)	47 (68)	41 (64)	32 (68)	0.05
Depression (F32 & F33)	11 (38)	15 (22)	8 (12)	10 (21)	
Anxiety (F41)	4 (14)	7 (10)	15 (23)	5 (11)	
**Baseline Score**,** mean (SD)**					
GSE^3^	22 (6)	27 (6.4)	24.1 (5.9)	28.8 (4.7)	< 0.01
EQ-5D Values Sweden	0.642 (0.111)	0.685 (0.137)	0.733 (0.132)	0.768 (0.128)	< 0.01
EQ-5D Values UK^4^	0.294 (0.233)	0.433 (0.290)	0.528 (0.276)	0.618 (0.271)	< 0.01
SMBQ^5^	5.8 (0.5)	5.5 (0.8)	5.3 (0.8)	4.6 (1.2)	< 0.01
PSS^6^	41.2 (5.2)	38.4 (6.3)	37.8 (6)	33 (6.3)	< 0.01
**Disposable Income**,** mean (SD)**					
Household^7^ (SEK^8^)	327 309 (128 787)	366 623 (272 897)	323 559 (100 910)	379 962 (166 448)	0.34

^1^SD: standard deviation, ^2^ICD: Internation Classification of Functioning, ^3^GSE: General Self-Efficacy, ^4^UK: United Kingdom, ^5^SMBQ: Shirom-Melamed Burnout Questionnaire, ^6^PSS: Perceived Stress Scale 14, ^7^Household: Disposable income per unit of consumption of a household, inclusive income earnt in another Nordic country, ^8^SEK: Swedish Crowns, ^*^Missing value

### Trajectory group costs

The *High Improvement Group* had the lowest mean cost for primary care (SEK 32 645), specialised outpatient care (SEK 19 574), prescription drugs (SEK 3 114) and productivity loss (SEK 123 812) amongst the trajectory groups (Table 4). Throughout all cost categories there was a sequential pattern of higher costs being associated with worse outcomes apart from the *Moderate Improvement Group* which had higher mean prescription drug costs (SEK 60 899) in comparison to all other groups.

**Table 4:** Mean Cost per Trajectory Group

Cost Type	No Improvement	Low Improvement	Moderate Improvement	High Improvement
	**(n = 29)**	**(n = 69)**	**(n = 64)**	**(n = 47)**
**Primary Care**, Mean Cost, SEK^1^ (CI^2^)	77 798 (59 024, 96 572)	55 426 (46 408, 64 445)	45 746 (38 457, 53 035)	32 645 (25 384, 39 906)
**Specialised Outpatient Care** Mean Cost, SEK (CI)	69 287 (20 484, 118 090)	46 455 (23 834, 69 075)	33 440 (21 756, 45 123)	19 574 (12 745, 26 402)
**Prescription Drugs**, Mean Cost, SEK (CI)	20 594 (1 102, 40 086)	12 352 (2 984, 21 720)	60 899 (-44 040, 165 837)	3 114 (2 109, 4 118)
**Productivity Loss**, Mean Cost, SEK (CI)	330 104 (200 298, 459 909)	235 807 (170 951, 300 662)	184 038 (133 052, 235 024)	123 812 (76 376, 171 249)
**Intervention Costs**, Mean Cost, SEK (CI)	678 (348, 1 008)	388 (263, 514)	355 (234, 475)	419 (286, 552)

^1^SEK: Swedish Crown, ^2^CI: Confidence Interval

#### Two year CCA results

Distribution of participants across the four trajectory groups was similar between the intervention and control groups, with a slightly greater proportion of participants in the *High Improvement Group* being from the intervention arm, 25% compared with 21% in the control group (Table 5). In terms of healthcare utilisation and associated costs, the intervention group had a lower mean difference in primary care (SEK − 3 454), Prescription Drugs (SEK − 35 081) and Productivity Loss (SEK − 10 711), however, a higher mean difference in Specialised Outpatient Care (SEK 7 687). Based on single imputation, QALY values were slightly lower at two years among those receiving the intervention using the UK value set (1.19 vs. 1.3) but were the same based on the Swedish value set (1.61 vs. 1.61). Multiple imputations set at 20 and 100 yielded similar results, with the Swedish value set having higher scores in the intervention group, 1.62 vs. 1.61 and 1.63 vs. 1.61, respectively.

**Table 5:** Cost-Consequence Results

Category	Intervention (*n* = 102)	Control (*n* = 107)	Difference	*p*-value
Groups, *n* (%)				
High Improvement Group	25 (25%)	22 (21%)	**+ 4 pp** ^**1**^	0.66
Moderate Improvement Group	31 (30%)	33 (31%)	**– 1 pp**	0.8
Low Improvement Group	32 (31%)	35 (33%)	**– 2 pp**	0.55
No Improvement Group	14 (14%)	15 (14%)	**0 pp**	0.81
**Costs**,** mean SEK**^**2**^**(CI**^**3**^**)**				
Primary Care Mean Cost, SEK (CI)	48 675 (41 197, 56 154)	52 129 (45 039, 59 218)	**– 3 454**	0.51
Specialised Outpatient Care Mean Cost, SEK (CI)	43 528 (24 463, 62 592)	35 841 (24 643, 47 039)	**7 687**	0.49
Prescription Drugs Mean Cost, SEK (CI)	8 324 (4 154, 12 494)	43 405 (–19 684, 106 494)	**– 35 081**	0.28
Productivity Loss Mean Cost, SEK (CI)	202 370 (153 192, 251 548)	213 081 (164 188, 261 973)	**– 10 711**	0.76
Intervention Costs* Mean Cost, SEK (CI)	871 (765, 977)	–	–	–
**UK** ^**4**^ **QALY** ^**5**^ **(single imp.** ^**6**^ **)**	1.19 (1.09, 1.29)	1.3 (1.23, 1.38)	**− 0.11**	0.08
**Swedish QALY (single imp.)**	1.61 (1.57, 1.64)	1.61 (1.57, 1.65)	**0**	0.88
**UK QALY** **(20 imp.)**	1.26 (1.24, 1.28)	1.33 (1.32, 1.35)	**– ** **0.07**	0.42
**Swedish QALY (20 imp.)**	1.62 (1.62, 1.63)	1.61 (1.6, 1.62)	**+ 0.01**	0.58
**UK QALY** **(100 imp.)**	1.27 (1.26, 1.28)	1.33 (1.32, 1.34)	**–** ** 0.06**	0.44
**Swedish QALY (100 imp.)**	1.63 (1.63, 1.63)	1.61 (1.6, 1.61)	**+ 0.02**	0.46

^1^pp: Percentage Points, ^2^SEK: Swedish Crowns, ^3^CI: Confidence Interval, ^4^UK: United Kingdom, ^5^QALY: Quality-Adjusted, ^6^imp:imputations

### Sensitivity analysis

Sensitivity analysis using the Swedish experience-based value set resulted in two trajectory groups (Supplementary Fig. 2). Group one (*n =* 92) is characterised by improvements in all outcome measures beyond the aforementioned clinical thresholds and is 53% intervention group participants. Group two (*n* = 117) exhibit small improvements, although, GSE is unchanged and participants remained above thresholds for moderate stress and clinical burnout. Comparison of EQ-5D values shows that results are more tightly grouped for the Swedish experience-based value set, indicating a rationale for fewer groups than the UK preference-based value set (Supplementary Fig. 3a and 3b).

## Discussion

The results of this study, which evaluated a PCC add-on intervention for people with CMD, were cost-saving, with the intervention group having lower total and mean costs for primary care, prescription drugs and productivity loss. The GBTM identified four trajectory groups: *High*,* Moderate*,* Low*, and *No Improvement* over a two-year time horizon. Demographics, such as education and civil status, as well as a primary diagnosis of depression and worse baseline scores for the self-reported outcome measures were key indicators for determining the longitudinal health improvement/deterioration of people with CMD. The results, however, showed no statistically significant differences between intervention and control groups with respect to trajectory group division.

Decision-making within healthcare incurs an opportunity cost; treating one individual means that those resources are no longer available to treat another [35]. Therefore, a single summary figure of health, such as EQ-5D values estimated using the EQ-5D, can be used as a useful tool when comparing the benefits of different treatments. Despite only incorporating one item related to mental health (Depression and Anxiety), the EQ-5D has been shown to adequately capture the symptoms related to CMD [36]. The GBTM results support this, with changes in EQ-5D values following the same longitudinal trajectories as GSE, SMBQ and PSS for each of the four trajectory groups. However, Brazier et al. [37] highlight that the intersectionality of mental health, which stretches across multiple social domains, requires greater information in capturing the needs of CMD patients beyond that afforded by the EQ-5D. Although multiple outcome measures would be more appropriate in these instances, without preference weighting such as that used when calculating QALYs, decision makers face problems with making comparisons across all sectors or combining them to provide an overall measure of benefit.

Further insight into summary measures in health economics, such as the Incremental Cost-Effectiveness Ratio (ICER), highlights a problem when evaluating the effectiveness of interventions tailored towards CMD. The ICER is a ratio representing the difference in costs and the difference in health outcomes, and at one year in the PROMISE study showed the intervention to be both more effective and less costly (i.e. dominant) [9]. This favourable result could, in turn, lead to decision makers implementing the PCC add-on intervention as a treatment for all patients with CMD without appropriate consideration of the aforementioned trajectory group differences. Such a decision could prove problematic considering that 45% of the intervention group had either low or no improvement, therefore requiring either more or different care to meet their individual needs. Herein lies the opportunity to combine health economics with methods for combining multiple outcome measures, such as GBTM or multi-criteria decision analysis, to further understand the individual needs of patients that move beyond group mean estimates.

Adequately addressing the large variety of social determinants that influence population and patient health has often been cited as a pitfall in traditional health economic methods, despite factors such as education level, income, and living standards being associated with increased morbidity and mortality [38]. The *No Improvement Group* had the lowest percentage of participants with a university education and the greatest percentage of people living alone and with a primary diagnosis of depression. They also experienced the worst baseline health with respect to each of the four outcome measures. This aligns with previous research [39, 40] that has shown that people living alone and with lower education can have higher prevalence of anxiety/depression and higher SMBQ scores. Based on the results of the GBTM, people with these social determinants also responded less well irrespective of being treated with the intervention or usual care. This emphasises both a greater need to incorporate social determinants into the planning of interventions, and the importance of additional or tailored support to achieve meaningful improvements for these patients.

The intersectoral nature of CMDs with respect to resource use is a problem that extends beyond healthcare costs. Lathe et al. [35] highlight the failings of previous research that has followed narrow healthcare or payer perspectives with respect to mental health, in that they bear only a fraction of the total costs. Reporting of costs from a societal perspective, including those costs associated with productivity loss, or more specifically in the case of this analysis, absenteeism, goes some way to alleviating this problem. However, it should be noted that this still leaves significant gaps in capturing the intersectoral spillovers associated with poor mental health. An accurate representation of costs should account for presenteeism, which has been shown to improve at a greater rate alongside improvements in mental health, as well as addressing social domains (social functioning and participation) [41]. Not only have these spillover effects been advocated for in mental health interventions but they have also been identified as a key criterion when evaluating PCC interventions [6].

### Strengths and limitations

The strengths of the study are the use of a randomised controlled trial design that reduces the risk of bias [35], and the use of near-complete registers at both the national and regional level that provided a detailed overview of the costs and effects of illness. Furthermore, through the use of GBTM, likely trajectories of individual health and their expected costs and outcomes can facilitate modifications to early interventions and harm reduction, helping to avoid care that may prove less effective.

GBTM can present analytic limitations by identifying spurious subgroups, especially when there are no trajectories in the data, when individual trajectories are a single continuum of values of the variable under study or when individual trajectories have the same shape or are distributed on a continuum around the mean trajectory [42]. However, Nagin et al. [5] address this as a misaligned interpretation with respect to the objective when using multi-trajectory modelling. The true number of groups is a quixotic figure, as the model is merely an approximation of the unknown distribution of measurements over time. Therefore, GBTM is not intended to identify the true number of groups, but rather to identify distinct features of the data. What is evident, and a strength of this study, is that the consistency across multiple outcome measures supports the validity of the trajectory groups and suggests that interventions could be more effectively tailored based on predicted patient trajectories.

Further limitations within the study are related to missing values in the self-reported outcomes measures used in the GBTM. Firstly, both the intervention and control groups had missing data on all outcomes at each of the measurement points, except complete data for GSE based on imputing the mean value for participants who had scored seven or more items on the scale [18]. To adjust for this, advanced imputation methods and bootstrapping were applied to the data to address uncertainty in the final effect estimates before being applied to the multi-trajectory model. It is important to note that GBTM calculation only permits a single imputed datafile. Although this may reduce the precision of inference, the underlying mean differences remain somewhat stable, an important factor for GBTM [43]. Multiple imputation, despite not being appropriate for GBTM, notably affected the final QALY results. However, it is outside the scope of this paper to further evaluate the use of imputation.

In the PROMISE study, several additional secondary outcome measures (e.g. Hospital Anxiety and Depression Scale, Multidimensional Fatigue Inventory) were used. However, these outcomes were placed later in the questionnaire order due to their perceived relevance by the research team and had poorer response frequencies. Consequently, these questionnaires were not connected to the patient register and could therefore not be evaluated.

### Recommendations for future research

Although statistical power was not initially calculated for more advanced subgroup analyses within the trajectory groups, descriptively, the data highlight that people with poorer baseline outcomes and specific social determinants responded considerably worse to both PCC and usual care. The results support the routine use of social and health indicators in identifying individuals at elevated risk who could benefit from additional support and incorporating them into the design and implementation of PCC interventions. This further highlights the importance of stratification approaches in future studies to ensure that the results (costs and effects) are accurately representative of the subgroup populations.

## Conclusion

This study shows that combining GBTM with cost-consequence analysis provides a practical and informative approach for evaluating person-centred, complex interventions. Although the PCC add-on was delivered at lower overall cost, substantial variation in individual response patterns underscores the importance of recognising patient heterogeneity when interpreting outcomes. Distinct trajectories were strongly linked to baseline health and social determinants, indicating that more targeted or tailored support may be required for those at greatest risk of poor improvement. Integrating trajectory-based methods into future evaluations can support more nuanced decision-making informing the effective resource allocation for people with CMD.

## Supplementary Information

Below is the link to the electronic supplementary material.

Supplementary Material 1

## Data Availability

The datasets generated and analysed during the current study are not publicly available because they contain information that can compromise research participants’ privacy/consent. Such data can only be made available, after legal review, to researchers who meet the criteria to access such sensitive and confidential data, according to the General Data Protection Regulation, the Swedish Data Protection Act, the Swedish Ethical Review Act, and the Swedish Public Access to Information and Secrecy Act. Readers may contact Professor Andreas Fors (andreas.fors@fhs.gu.se) regarding this data.

## Abbreviations

PCC: Person–centred Care
GBTM: Group–based Trajectory Modelling
HCP: Healthcare Professional
GSE: General Self–Efficacy
CMD: Common Mental Disorders
CCA: Cost–Consequence Analysis
ICD: International Classification of Disease
PSS: Perceived Stress Scale
SMBQ: Shirom Melamed Burnout Questionnaire
DRG: Diagnose Related Groups
NBHW: National Board of Health and Welfare
MIDAS: Micro Data for Analysis of the Social Insurance System
QALY: Quality Adjusted Life Years
UK: United Kingdom
APP: Average Posterior Probability
SEK: Swedish Crowns
ICER: Incremental Cost–Effectiveness Ratio
