# Supplementary material for: Group-based trajectory modelling of multiple health outcomes in a cost–consequence framework: a randomized controlled trial of a remote person-centred care intervention for people with common mental disorders in Sweden
Source: J Patient Rep Outcomes. 2026 Mar 20;10:54. doi: 10.1186/s41687-026-01043-y (PMC13066024; doi:10.1186/s41687-026-01043-y)
Supplement: Supplementary file 1 — Supplementary Material 1 [file 41687_2026_1043_MOESM1_ESM.docx]

Group-Based Trajectory Modelling of Multiple Health Outcomes in a Cost–Consequence Framework: A Randomized Controlled Trial of a Remote Person-Centred Care Intervention for People with Common Mental Disorders in Sweden

Table of Contents

[Supplementary Figure 1. Consort Flow Chart 3](#_Toc221277226)

[Supplementary Table 1. Unit Costs 3](#_Toc221277227)

[Supplementary Table 2. Missing Cost Values 4](#_Toc221277228)

[Supplementary Table 3. Internal consistency of study measures 5](#_Toc221277229)

[Supplementary Table 4. Non-Imputed and Imputed Outcome Scores 6](#_Toc221277230)

[Supplementary Table 5. Trajectory Analysis Results 8](#_Toc221277231)

[GBTM Model Fit Process 8](#_Toc221277232)

[Supplementary Table 6. Post-Hoc Statistical Differences 10](#_Toc221277233)

[Supplementary Figure 2. Trajectory Groups (Swedish Experience-Based Value Set) 11](#_Toc221277234)

[Supplementary Figure 3a. Comparison of HRQoL – UK Preference-Based Value Set 12](#_Toc221277235)

[Supplementary Figure 3b. Comparison of HRQoL – Swedish Experience-Based Value 12](#_Toc221277236)

[Set 12](#_Toc221277237)

[Consort Checklist 13](#_Toc221277238)

[Cheers Checklist 16](#_Toc221277239)

[References 20](#_Toc221277240)

**Not eligible (n=315)**

Did not meet the inclusion criteria **(n=315)**

**Invited to participate in the study (n=787)**

Nonrespondents (n=199)

Declined to participate (n=588)

**Enrolled**

**Assessed for eligibility (n=1317)**

**Randomised (n=215)**

**Control Group**

**(n=108)**

**Intervention Group (n=107)**

**Lost to follow-up:**

Withdrew (n=5)

**Missing data:**

Complete missing (n= 0)

Partial missing (n=11)

**Lost to follow-up:**

Withdrew (n=1)

**Missing data:**

Complete missing (n=0)

Partial missing (n=12)

**Baseline**

**3 months**

**Missing data:**

Complete missing (n=18)

Partial missing (n=11)

**Missing data:**

Complete missing (n=23)

Partial missing (n=10)

**Missing data:**

Complete missing (n=19)

Partial missing (n=11)

**6 months**

**Missing data:**

Complete missing (n=11)

Partial missing (n=11)

**Missing data:**

Complete missing (n=18)

Partial missing (n=13)

**Missing data:**

Complete missing (n=16)

Partial missing (n=14)

**12 months**

**Missing data:**

Complete missing (n=24)

Partial missing (n=5)

**Missing data:**

Complete missing (n=20)

Partial missing (n=15)

**18 months**

**24 months**

**Missing data:**

Complete missing (n=25)

Partial missing (n=4)

**Lost to follow-up:**

Deceased (n=1)

**Missing data:**

Complete missing (n=23)

Partial missing (n=10)

**Intervention Group**

**Analysed (n=102)**

**Control Group Analysed (n=107)**

# **Supplementary Figure 1. Consort Flow Chart**

| **Care Type** | **Care Provider** | **Unit Cost (SEK^1^)** | **Intervention** | | **Control** | |
| --- | --- | --- | --- | --- | --- | --- |
|  |  |  | **Contact Frequency** | **Mean Cost**  **(CI^2^)** | **Contact Frequency** | **Mean Cost**  **(CI)** |
| **Primary Care [1]** | Physician visit | 2 442 | 946 | 23 342  (20 381, 26 821) | 1 067 | 23 621  (20 367, 27 008) |
|  | Physician telephone | 814 | 228 | 2 466  (2 109, 2 877) | 302 | 2 741  (2 305, 3 186) |
|  | Nurse visit | 977 | 1012 | 9 784  (7 860, 12 569) | 1 240 | 11 749  (9 987, 13 976) |
|  | Nurse telephone | 326 | 245 | 1 090  (894, 1 357) | 206 | 1 033  (822, 1 303) |
|  | Physio^3^. & Occ^4^. Therapist visit | 977 | 781 | 12 333  (9 570, 15 476) | 809 | 11 793  (8 775, 16 020) |
|  | Physio. & Occ. Therapist telephone | 326 | 33 | 613  (427, 929) | 25 | 464  (372, 539) |
|  | Others visit | 977 | 710 | 10 041  (7 891, 12 756) | 738 | 8 965  (7 471, 10 629) |
|  | Others telephone | 326 | 268 | 1 365  (983, 2 155) | 311 | 1 276  (1 034, 1 591) |
|  | **Total** |  | **4223** | **78 999**  **(77 688, 80 512)** | **4698** | **78 616**  **(77 378, 79 845)** |
| **Specialised Outpatient Care [2]** | Physician visit |  | 455 | 4 389  (4 129, 4 750) | 357 | 4 533  (4 239, 4 938) |
|  | Nurse visit |  | 234 | 3 428  (3 152, 3 764) | 142 | 2 520  (2 379, 2 673) |
|  | Physio. & Occ. Therapist visit |  | 108 | 2 279  (2 192, 2 370) | 12 | 2 153  (1 927, 2 366) |
|  | Others visit |  | 180 | 2 660  (2 564, 2 758) | 146 | 2 703  (2 606, 2 799) |
|  | Polyclinic |  |  |  | 3 | 49 645  (42 201, 60 812) |
|  | Hospitalisation |  | 20 | 45 783  (37 904, 55 334) | 25 | 57 533  (47 864, 68 547) |
|  | **Total** |  | **997** | **215 011**  **(201 109, 228 536)** | **685** | **110 516**  **(104 041, 118 150)** |

# **Supplementary Table 1. Unit Costs**

^1^ SEK: Swedish Crowns, ^2^ CI: Confidence Interval, ^3^ Physio: Physiotherapist, ^4^ Occ: Occupational Therapist

| **Supplementary Table 2. Missing Cost Values** | | | | | | |
| --- | --- | --- | --- | --- | --- | --- |
| **Care Type** | **Care Provider** | | **Complete** | **Somatic** | **Median Imputation** | **Total** |
| **Primary Care** | | Physician Visit | 1,050 | 0 | 0 | 1,050 |
|  |  | Physician Telephone | 379 | 0 | 0 | 379 |
|  |  | Nurse Visit | 1,819 | 0 | 0 | 1,819 |
|  |  | Nurse Telephone | 390 | 0 | 0 | 390 |
|  |  | Physio^1^. & Occ^2^.  Therapist Visit | 1,099 | 0 | 0 | 1,099 |
|  |  | Physio. & Occ.  Therapist Telephone | 41 | 0 | 0 | 41 |
|  |  | Other Visit | 170 | 0 | 0 | 170 |
|  |  | Other Telephone | 377 | 0 | 0 | 377 |
| **Specialised Outpatient Care** | | Physician Visit | 455 | 1 | 234 | 690 |
|  |  | Nurse Visit | 117 | 0 | 0 | 117 |
|  |  | Physio. & Occ.  Therapist Visit | 71 | 0 | 14 | 85 |
|  |  | Other Visit | 60 | 0 | 47 | 107 |
|  |  | Polyclinical | 18 | 0 | 3 | 21 |
|  |  | Hospitalisation | 36 | 0 | 2 | 38 |
| **Total** |  | | **6,098** | **1** | **394** | **6,493** |

^1^ Physio: Physiotherapist, ^2^ Occ: Occupational Therapist

#

# **Supplementary Table 3. Internal consistency of study measures**

| **Self-reported Outcome Measure** | **Items (n)** | **Sample size (n)** | **Cronbach’s α** | **95% CI** |
| --- | --- | --- | --- | --- |
| General Self-Efficacy Scale (GSE) | 10 | 206–209 | 0.91 | [.90, .93] |
| Perceived Stress Scale (PSS) | 14 | 207–209 | 0.78 | [.73, .82] |
| Shirom–Melamed Burnout Questionnaire (SMBQ) | 22 | 200–209 | 0.95 | [.93, .96] |
| **Note.** Cronbach’s alpha values are raw coefficients. Confidence intervals are based on Feldt’s method. Missing data were handled using pairwise deletion. | | | | |

# **Supplementary Table 4. Non-Imputed and Imputed Outcome Scores**

|  |  | **Non-imputed mean values** | | **Missing Data** | | **Single Imputation** | | **Multiple Imputation (20)** | |  |
| --- | --- | --- | --- | --- | --- | --- | --- | --- | --- | --- |
|  |  |  |  |  |  |  |  |  |  |  |
|  |  | **Control** | **Intervention** | **Control** | **Intervention** | **Control** | **Intervention** | **Control** | **Intervention** |  |
| **Variable** | **Time** | **Mean(CI^1^)** | **Mean (CI)** | **(n = 107)** | **(n = 102)** | **Mean (CI)** | **Mean (CI)** | **Mean (CI)** | **Mean (CI)** |  |
| **Utility Values UK^2^** |  |  |  |  |  |  |  |  |  |  |
|  | Baseline | 0.463 (0.408, 0.517) | 0.506 (0.447, 0.564) | 3 (2.8%) | 2 (2.0%) | 0.464 (0.408, 0.514) | 0.506 (0.448, 0.564) | 0.464 (0.408, 0.514) | 0.506 (0.448, 0.564) |  |
|  | 3-months | 0.635 (0.576, 0.691) | 0.648 (0.586, 0.705) | 26 (24.3%) | 19 (18.6%) | 0.636 (0.587, 0.683) | 0.650 (0.599, 0.703) | 0.627 (0.616, 0.638) | 0.637 (0.624, 0.649) |  |
|  | 6-months | 0.678 (0.627, 0.733) | 0.703 (0.643, 0.756) | 11 (10.3%) | 20 (19.6%) | 0.697 (0.649, 0.746) | 0.710 (0.663, 0.755) | 0.679 (0.668, 0.689) | 0.701 (0.690, 0.712) |  |
|  | 12-months | 0.745 (0.695, 0.790) | 0.713 (0.654, 0.768) | 16 (15.0%) | 18 (17.6%) | 0.750 (0.708, 0.793) | 0.710 (0.662, 0.761) | 0.740 (0.730, 0.750) | 0.713 (0.703, 0.724) |  |
|  | 18-months | 0.719 (0.660, 0.776) | 0.745 (0.685, 0.801) | 20 (18.7%) | 24 (23.5%) | 0.722 (0.672, 0.770) | 0.762 (0.711, 0.807) | 0.722 (0.712, 0.733) | 0.753 (0.742, 0.764) |  |
|  | 24-months* | 0.716 (0.659, 0.771) | 0.707 (0.637, 0.772) | 24 (22.4%) | 25 (24.5%) | 0.707 (0.659, 0.752) | 0.702 (0.649, 0.755) | 0.710 (0.699, 0.721) | 0.704 (0.692, 0.717) |  |
| **Utility Values SWE^3^** |  |  |  |  |  |  |  |  |  |  |
|  | Baseline | 0.707 (0.681, 0.733) | 0.718 (0.691, 0.744) | See above | See Above | 0.707 (0.679, 0.736) | 0.718 (0.692, 0.743) | 0.707 (0.679, 0.736) | 0.718 (0.692, 0.743) |  |
|  | 3-months | 0.790 (0.761, 0.820) | 0.805 (0.778, 0.831) |  |  | 0.792 (0.767, 0.818) | 0.799 (0.775, 0.823) | 0.788 (0.782, 0.793) | 0.800 (0.795, 0.805) |  |
|  | 6-months | 0.819 (0.791, 0.845) | 0.838 (0.813, 0.862) |  |  | 0.828 (0.801, 0.853) | 0.839 (0.820, 0.861) | 0.819 (0.813, 0.824) | 0.837 (0.832, 0.842) |  |
|  | 12-months | 0.860 (0.837, 0.882) | 0.843 (0.815, 0.869) |  |  | 0.858 (0.834, 0.878) | 0.841 (0.817, 0.865) | 0.860 (0.856, 0.865) | 0.841 (0.836, 0.846) |  |
|  | 18-months | 0.845 (0.816, 0.872) | 0.860 (0.831, 0.885) |  |  | 0.838 (0.811, 0.864) | 0.863 (0.842, 0.883) | 0.846 (0.840, 0.851) | 0.863 (0.858, 0.868) |  |
|  | 24-months* | 0.855 (0.824, 0.881) | 0.842 (0.807, 0.872) |  |  | 0.844 (0.815, 0.870) | 0.838 (0.809, 0.865) | 0.853 (0.847, 0.858) | 0.837 (0.831, 0.843) |  |
| **GSE^4^** |  |  |  |  |  |  |  |  |  |  |
|  | Baseline | 25.91 (24.80, 27.15) | 25.78 (24.47, 26.99) | 0 (0.0%) | 0 (0.0%) | 25.91 (24.8, 27.09) | 25.78 (24.54, 26.97) | 25.91 (24.8, 27.09) | 25.78 (24.54, 26.97) |  |
|  | 3-months | 25.92 (24.56, 27.29) | 27.84 (26.66, 29.05) | 23 (21.5%) | 18 (17.6%) | 26.28 (25.1, 27.47) | 27.14 (25.91, 28.23) | 26.02 (25.75, 26.29) | 27.66 (27.38, 27.91) |  |
|  | 6-months | 27.62 (26.41, 28.78) | 28.70 (27.35, 30.09) | 13 (12.1%) | 20 (19.6%) | 27.37 (26.25, 28.51) | 28.46 (27.14, 29.66) | 27.52 (27.28, 27.77) | 28.70 (28.43, 29.00) |  |
|  | 12-months | 28.80 (27.61, 29.99) | 30.21 (28.89, 31.57) | 18 (16.8%) | 18 (17.6%) | 28.66 (27.49, 29.75) | 29.92 (28.71, 31.06) | 28.89 (28.64, 29.14) | 30.25 (29.97, 30.52) |  |
|  | 18-months | 29.13 (27.68, 30.53) | 29.58 (28.09, 30.95) | 23 (21.5%) | 24 (23.5%) | 28.87 (27.49, 30.04) | 29.62 (28.37, 30.83) | 29.18 (28.91, 29.47) | 29.41 (29.14, 29.69) |  |
|  | 24-months* | 29.40 (27.93, 30.90) | 30.22 (28.74, 31.77) | 22 (20.6%) | 25 (24.5%) | 28.81 (27.38, 30.19) | 29.98 (28.6, 31.27) | 29.25 (28.95, 29.55) | 29.86 (29.52, 30.17) |  |
| **SMBQ^5^** |  |  |  |  |  |  |  |  |  |  |
|  | Baseline | 5.23 (5.03, 5.42) | 5.34 (5.14, 5.53) | 9 (8.4%) | 8 (7.8%) | 5.24 (5.04, 5.41) | 5.34 (5.15, 5.49) | 5.24 (5.04, 5.41) | 5.34 (5.15, 5.49) |  |
|  | 3-months | 4.67 (4.36, 5) | 4.47 (4.21, 4.73) | 32 (29.9%) | 26 (25.5%) | 4.67 (4.44, 4.91) | 4.43 (4.24, 4.63) | 4.34 (4.31, 4.38) | 4.15 (4.12, 4.18) |  |
|  | 6-months | 4.33 (4.04, 4.62) | 4.05 (3.75, 4.34) | 19 (17.8%) | 24 (23.5%) | 4.32 (4.1, 4.57) | 4.04 (3.81, 4.29) | 4.08 (4.04, 4.11) | 3.89 (3.85, 3.93) |  |
|  | 12-months | 3.91 (3.56, 4.21) | 3.97 (3.66, 4.28) | 28 (26.2%) | 28 (27.5%) | 3.91 (3.65, 4.19) | 3.95 (3.71, 4.21) | 3.83 (3.79, 3.87) | 3.91 (3.87, 3.94) |  |
|  | 18-months | 3.89 (3.58, 4.21) | 3.89 (3.6, 4.22) | 31 (29.0%) | 29 (28.4%) | 3.86 (3.59, 4.13) | 4.05 (3.76, 4.31) | 3.82 (3.78, 3.85) | 3.79 (3.75, 3.82) |  |
|  | 24-months* | 3.87 (3.5, 4.23) | 3.7 (3.33, 4.06) | 30 (28.0%) | 27 (26.5%) | 3.79 (3.5, 4.07) | 3.69 (3.38, 4.01) | 3.78 (3.74, 3.82) | 3.75 (3.71, 3.79) |  |
| **PSS^6^** |  |  |  |  |  |  |  |  |  |  |
|  | Baseline | 37.59 (36.45, 38.87) | 37.22 (35.77, 38.66) | 2 (1.9%) | 2 (2.0%) | 37.59 (36.4, 38.75) | 37.22 (35.9, 38.57) | 37.59 (36.4, 38.75) | 37.22 (35.9, 38.57) |  |
|  | 3-months | 31.11 (29.19, 33.14) | 29.15 (27.07, 31.3) | 25 (23.4%) | 21 (20.6%) | 30.98 (29.26, 32.67) | 29.20 (27.19, 31.19) | 32.13 (31.91, 32.32) | 32.46 (32.28, 32.64) |  |
|  | 6-months | 28.17 (26.2, 30.06) | 26.71 (24.78, 28.72) | 14 (13.1%) | 26 (25.5%) | 28.38 (26.54, 30.24) | 25.82 (23.93, 27.33) | 32.94 (32.77, 33.09) | 32.79 (32.62, 32.98) |  |
|  | 12-months | 25.99 (23.85, 28.12) | 25.25 (22.68, 27.8) | 20 (18.7%) | 23 (22.5%) | 26.48 (24.72, 28.43) | 25.04 (22.99, 27.15) | 33.07 (32.92, 33.22) | 32.51 (32.35, 32.67) |  |
|  | 18-months | 26.51 (24.01, 28.83) | 25.74 (23.44, 27.99) | 28 (26.2%) | 24 (23.5%) | 26.69 (24.69, 28.89) | 26.66 (24.62, 28.81) | 32.08 (31.93, 32.22) | 32.16 (31.97, 32.34) |  |
|  | 24-months* | 26.23 (23.91, 28.75) | 24.12 (21.35, 26.92) | 23 (21.5%) | 27 (26.5%) | 26.34 (24.18, 28.58) | 24.08 (21.83, 26.41) | 32.09 (31.88, 32.30) | 32.16 (32.00, 32.32) |  |

^1^ CI: Confidence Interval, ^2^ UK: United Kingdom, ^3^ SWE: Swedish, ^4^ GSE: General Self-Efficacy, ^5^ SMBQ: Shirom-Melamed Burnout Questionnaire, ^6^ PSS: Perceived Stress Scale, ^*^Missing value

# **Supplementary Table 5. Trajectory Analysis Results**

| **Trajectory Group** | **Count** | **Mean Posterior Probability** | **Observed Outcome** | **Predicted Outcome** | **Proportion** | **Total Probability** |
| --- | --- | --- | --- | --- | --- | --- |
| Low | 69 | 0.9621 | 51.4381 | 49.5307 | 0.3301 | 0.3386 |
| High | 47 | 0.9752 | 135.4479 | 135.8819 | 0.2249 | 0.2243 |
| Moderate | 64 | 0.9179 | 25.3209 | 26.5042 | 0.3062 | 0.2966 |
| No | 29 | 0.9942 | 1055.653 | 1040.263 | 0.1388 | 0.1405 |

**Mean Posterior Probability:** The average probability that individuals truly belong to their assigned group; higher values indicate better classification accuracy, **0.70** = acceptable, **0.80** = good, **0.90** = excellent. **Observed Outcome**: The actual mean score of the measured outcome for individuals assigned to the group. **Predicted Outcome**: The model-estimated mean outcome for the group, derived from the fitted trajectory function. Close alignment with “Observed Outcome” indicates a well-fitting model. **Proportion**: The observed proportion of the sample assigned to each trajectory group (Count ÷ Total sample). **Total Probability**: The model-estimated population probability for each trajectory group (i.e., expected group size). Should be close to the “Proportion” column; values within ±0.05 are generally considered acceptable [3]**.**

# **GBTM Model Fit Process**

Trajectory groups for each of the four outcomes (GSE, utility values, PSS, and SMBQ) were estimated using an iterative model selection process. Analyses began by fitting linear trajectories, defined as straight lines indicating a constant rate of change over time. Models were initially estimated with two trajectory groups and then sequentially expanded to three, four, and five groups. Model fit was evaluated based on the proximity of the estimated mean values at each measurement point to the fitted trajectory, average posterior probability (APP) scores, and the size of each trajectory group. Linear specifications were assessed as inadequate due to substantial deviations between the estimated mean values and the fitted lines, as well as low APP scores.

The same procedure was then repeated using quadratic trajectories, which allow for curvature in the trajectory and changes in the rate of change over time. However, quadratic models were also deemed inappropriate, as the estimated mean values remained distant from the fitted trajectories and APP scores were low.

Finally, cubic trajectories, which allow for more flexible patterns with multiple changes in direction, were estimated. Under the cubic specification, a four-group solution provided the best model fit for each individual outcome, with the exception of GSE. The outcomes were subsequently combined and modelled using a cubic function, and the same iterative process was applied to determine the optimal number of trajectory groups. A four-group solution was selected for the combined model, as it demonstrated the best overall fit, the highest APP scores, and an appropriate distribution of participants across groups.

**Supplementary Table 6. Post-Hoc Statistical Differences**

| **Outcome** | **Group**  ***p*-value** | **Groups** | **Individual**  ***p*-value** |
| --- | --- | --- | --- |
| **Education level** | 0.03 | moderate & low | 0.05 |
| **Diagnosis** | 0.05 | moderate & no | 0.03 |
| **GSE** | < 0.01 | moderate & **low** | 0.03 |
|  |  | no & **low** | < 0.01 |
|  |  | moderate & **high** | < 0.01 |
|  |  | no & **high** | < 0.01 |
| **HRQoL (Sweden)** | < 0.01 | **high** & low | < 0.01 |
|  |  | no & **high** | < 0.01 |
|  |  | no & **moderate** | < 0.01 |
| **HRQoL (UK)** | < 0.01 | **high** & low | < 0.01 |
|  |  | no & **high** | < 0.01 |
|  |  | no & **moderate** | < 0.01 |
| **SMBQ** | < 0.01 | **high** & low | < 0.01 |
|  |  | moderate & **high** | < 0.01 |
|  |  | no & **high** | < 0.01 |
|  |  | no & **moderate** | 0.03 |
| **PSS-14** | < 0.01 | **high** & low | < 0.01 |
|  |  | moderate & **high** | < 0.01 |
|  |  | no & **high** | < 0.01 |

^1^ GSE: General Self-Efficacy, ^2^ HRQoL: Health Related Quality of Life, ^3^ UK: United Kingdom, ^4^ SMBQ: Shirom-Melamed Burnout Questionnaire, ^4^ PSS-14: Perceived Stress Scale 14, **Bold Text:** Groups with better baseline score

# **Supplementary Figure 2. Trajectory Groups (Swedish Experience-Based Value Set)**


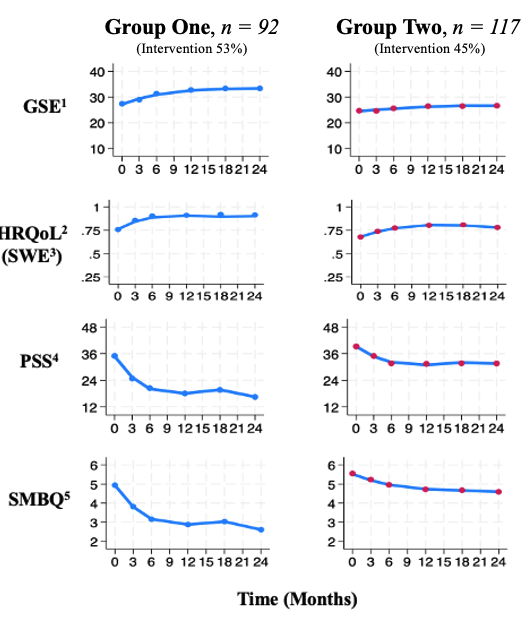


^1^General Self-Efficacy ^2^ HRQoL: Health Related Quality of Life, ^3^ SWE: Swedish, ^4^ PSS: Perceived Stress Scale, ^5^ SMBQ: Shirom-Melamed Burnout Questionnaire

# **Supplementary Figure 3a. Comparison of HRQoL – UK Preference-Based Value Set**


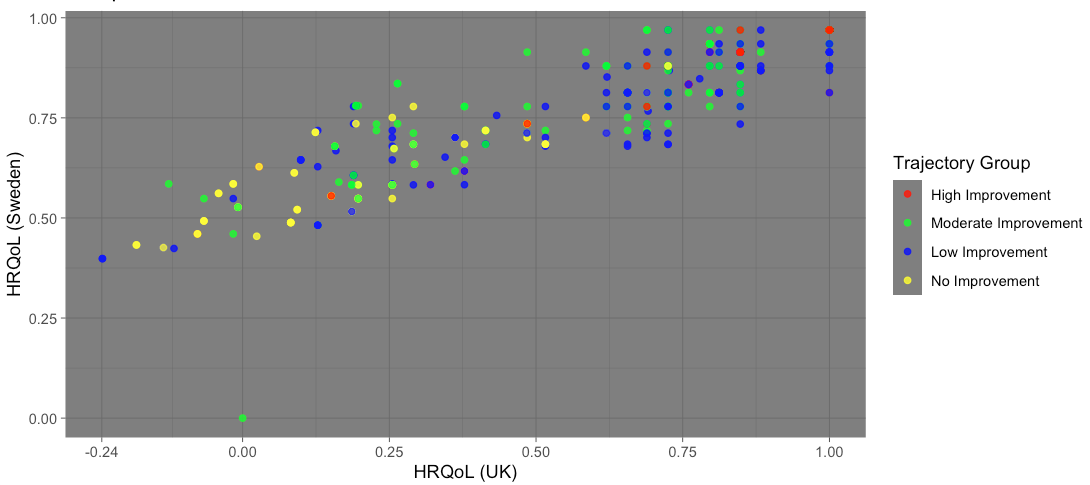


# **Supplementary Figure 3b. Comparison of HRQoL – Swedish Experience-Based Value**

# **Set**


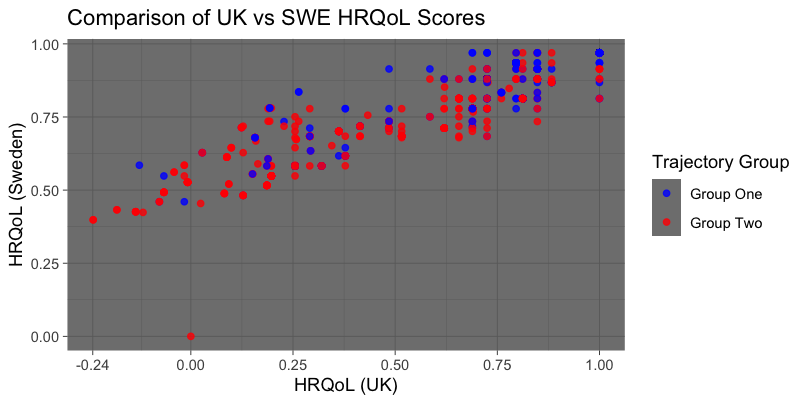


# **Consort Checklist**

|  | Section/topic | No | CONSORT 2025 checklist item description | Reported on page no. |
| --- | --- | --- | --- | --- |
|  | **Title and abstract** | | |  |
|  | Title and structured abstract | 1a | Identification as a randomised trial | 4 |
|  |  | 1b | Structured summary of the trial design, methods, results, and conclusions | 3 |
|  | **Open science** | | |  |
|  | Trial registration | 2 | Name of trial registry, identifying number (with URL) and date of registration | 31 |
|  | Protocol and statistical analysis plan | 3 | Where the trial protocol and statistical analysis plan can be accessed | 7 |
|  | Data sharing | 4 | Where and how the individual de-identified participant data (including data dictionary), statistical code and any other materials can be accessed | 31 |
|  | Funding and conflicts of interest | 5a | Sources of funding and other support (eg, supply of drugs), and role of funders in the design, conduct, analysis and reporting of the trial | 32 |
|  |  | 5b | Financial and other conflicts of interest of the manuscript authors | 32 |
|  | **Introduction** | | |  |
|  | Background and rationale | 6 | Scientific background and rationale | 5-6 |
|  | Objectives | 7 | Specific objectives related to benefits and harms | NA |
|  | **Methods** | | |  |
|  | Patient and public involvement | 8 | Details of patient or public involvement in the design, conduct and reporting of the trial | 7 |
|  | Trial design | 9 | Description of trial design including type of trial (eg, parallel group, crossover), allocation ratio, and framework (eg, superiority, equivalence, non-inferiority, exploratory) | 7-8 |
|  | Changes to trial protocol | 10 | Important changes to the trial after it commenced including any outcomes or analyses that were not prespecified, with reason | NA |
|  | Trial setting | 11 | Settings (eg, community, hospital) and locations (eg, countries, sites) where the trial was conducted | 7-8 |
|  | Eligibility criteria | 12a | Eligibility criteria for participants | 7-8 |
|  |  | 12b | If applicable, eligibility criteria for sites and for individuals delivering the interventions (eg, surgeons, physiotherapists) | NA |
|  | Intervention and comparator | 13 | Intervention and comparator with sufficient details to allow replication. If relevant, where additional materials describing the intervention and comparator (eg, intervention manual) can be accessed | 9 |
|  | Outcomes | 14 | Prespecified primary and secondary outcomes, including the specific measurement variable (eg, systolic blood pressure), analysis metric (eg, change from baseline, final value, time to event), method of aggregation (eg, median, proportion), and time point for each outcome | 11-12 |
|  | Harms | 15 | How harms were defined and assessed (eg, systematically, non-systematically) | NA |
|  | Sample size | 16a | How sample size was determined, including all assumptions supporting the sample size calculation | 8 |
|  |  | 16b | Explanation of any interim analyses and stopping guidelines | NA |
|  | Randomisation: |  |  |  |
|  | Sequence generation | 17a | Who generated the random allocation sequence and the method used | 8 |
|  |  | 17b | Type of randomisation and details of any restriction (eg, stratification, blocking and block size) | 8 |
|  |  |  |  | **Reported on page no.** |
|  | Allocation concealment mechanism | 18 | Mechanism used to implement the random allocation sequence (eg, central computer/telephone; sequentially numbered, opaque, sealed containers), describing any steps to conceal the sequence until interventions were assigned | 8 |
|  | Implementation | 19 | Whether the personnel who enrolled and those who assigned participants to the interventions had access to the random allocation sequence | 8 |
|  | Blinding | 20a | Who was blinded after assignment to interventions (eg, participants, care providers, outcome assessors, data analysts) | NA |
|  |  | 20b | If blinded, how blinding was achieved and description of the similarity of interventions | NA |
|  | Statistical methods | 21a | Statistical methods used to compare groups for primary and secondary outcomes, including harms | 13-14 |
|  |  | 21b | Definition of who is included in each analysis (eg, all randomised participants), and in which group | 13-14 |
|  |  | 21c | How missing data were handled in the analysis | 12 |
|  |  | 21d | Methods for any additional analyses (eg, subgroup and sensitivity analyses), distinguishing prespecified from post hoc | 14 |
|  | **Results** | | |  |
|  | Participant flow, including flow diagram | 22a | For each group, the numbers of participants who were randomly assigned, received intended intervention, and were analysed for the primary outcome | 8 / Supplementary figure 1. |
|  |  | 22b | For each group, losses and exclusions after randomisation, together with reasons | 8 |
|  | Recruitment | 23a | Dates defining the periods of recruitment and follow-up for outcomes of benefits and harms | 8 |
|  |  | 23b | If relevant, why the trial ended or was stopped | NA |
|  | Intervention and comparator delivery | 24a | Intervention and comparator as they were actually administered (eg, where appropriate, who delivered the intervention/comparator, how participants adhered, whether they were delivered as intended (fidelity)) | 9 |
|  |  | 24b | Concomitant care received during the trial for each group | NA |
|  | Baseline data | 25 | A table showing baseline demographic and clinical characteristics for each group | 16 |
|  | Numbers analysed,  outcomes and estimation | 26 | For each primary and secondary outcome, by group:  ● the number of participants included in the analysis  ● the number of participants with available data at the outcome time point  ● result for each group, and the estimated effect size and its precision (such as 95% confidence interval)  ● for binary outcomes, presentation of both absolute and relative effect size | 16-24 |
|  | Harms | 27 | All harms or unintended events in each group | NA |
|  | Ancillary analyses | 28 | Any other analyses performed, including subgroup and sensitivity analyses, distinguishing pre-specified from post hoc | 25 |
|  | **Discussion** | | |  |
|  | Interpretation | 29 | Interpretation consistent with results, balancing benefits and harms, and considering other relevant evidence | 26-28 |
|  | Limitations | 30 | Trial limitations, addressing sources of potential bias, imprecision, generalisability, and, if relevant, multiplicity of analyses | 28 |

# **Cheers Checklist**

| Topic  **Title** | No. | Item | is reported |
| --- | --- | --- | --- |
| Title  **Abstract** | 1 | Identify the study as an economic evaluation and specify the interventions being compared. | 1 |
| Abstract Introduction | 2 | Provide a structured summary that highlights context, key methods, results, and alternative analyses. | 2-3 |
| Background and  objectives  **Methods** | 3 | Give the context for the study, the study question, and its practical relevance for decision making in policy or practice. | 5-6 |
| Health economic analysis plan | 4 | Indicate whether a health economic analysis plan was developed and where available. | 7 |
| Study population | 5 | Describe characteristics of the study population (such as age range, demographics, socioeconomic, or clinical characteristics). | 7-8 |
| Setting and location | 6 | Provide relevant contextual information that may influence findings. | 7-8 |
| Comparators | 7 | Describe the interventions or strategies being  compared and why chosen. | 9 |
| Perspective | 8 | State the perspective(s) adopted by the study and why chosen. | 10 |
| Time horizon | 9 | State the time horizon for the study and why appropriate. | 10 |
| Discount rate | 10 | Report the discount rate(s) and reason chosen. | 10 |
| Selection of outcomes | 11 | Describe what outcomes were used as the measure(s) of benefit(s) and harm(s). | 11-12 |
| Measurement of outcomes | 12 | Describe how outcomes used to capture benefit(s) and harm(s) were measured. | 11-12 |
| Valuation of outcomes | 13 | Describe the population and methods used to measure and value outcomes. | 11-12 |
| Measurement and valuation of resources and costs | 14 | Describe how costs were valued. | 10-11 |
| Currency, price date, and conversion | 15 | Report the dates of the estimated resource quantities and unit costs, plus the currency and year of conversion. | 10-11 |
| Rationale and description of model | 16 | If modelling is used, describe in detail and  why used. Report if the model is publicly available and where it can be accessed. | NA |
| Analytics and assumptions | 17 | Describe any methods for analysing or statistically  transforming data, any extrapolation methods, and approaches for validating any model used. | 12-14 |
| Characterising heterogeneity | 18 | Describe any methods used for estimating how the results of the study vary for subgroups. | 13-14 |
| Characterising distributional effects | 19 | Describe how impacts are distributed across different individuals or adjustments made to reflect priority populations. | 13-14 |
| Characterising uncertainty | 20 | Describe methods to characterise any sources of uncertainty in the analysis. | 14 |
| Approach to engagement with patients and others affected by the study  **Results** | 21 | Describe any approaches to engage patients or service recipients, the general public, communities, or stakeholders (such as clinicians or payers) in the design of the study. | 9 |
| Study parameters | 22 | Report all analytic inputs (such as values, ranges, references) including uncertainty or distributional assumptions. | 15-23 |
| Summary of main  results | 23 | Report the mean values for the main categories of costs and outcomes of interest and summarise them in the most  appropriate overall measure. | 15-23 |
| Effect of uncertainty | 24 | Describe how uncertainty about analytic judgments, inputs, or projections affect findings. Report the effect of choice of discount rate and time horizon, if applicable. | 25 |
| Effect of engagement with patients and others  affected by the study | 25 | Report on any difference patient/service recipient, general public, community, or stakeholder involvement made to the approach or findings of the study | NA |
| Study findings, limitations, generalisability, and current knowledge  Other relevant information | 26 | Report key findings, limitations, ethical or equity considerations not captured, and how these could affect patients, policy, or practice. | 26-28 |
| Source of funding | 27 | Describe how the study was funded and any role  of the funder in the  identification, design, conduct, and reporting of the analysis | 32 |
| Conflicts of interest | 28 | Report authors conflicts of interest according to journal or International Committee of Medical Journal Editors requirements. | 32 |

# **References**

1. Swedish Association of Local Authorities and Regions. Statistik om hälso och sjukvård samt regional utveckling 2022 [Statistics on healthcare and regional development 2022]. [cited 2024 Mar 5]; https://https://skr.se/download/18.14fb7b721997a8f53f23d1c8/1758791429548/Statistik-om-halso-och-sjukvard-samt-regional-utveckling-2023-1010.pdf. Accessed 5 Mar 2024

2. National Board for Health and Welfare. Viktlistor för NordDRG [Weight list for NordDRG]. 2022 [cited 2023 June 14]; https://www.socialstyrelsen.se/statistik-och-data/klassifikationer-och-koder/drg/viktlistor/. Accessed 14 June 2023

3. Wheeler AP. Group based trajectory models in stata – some graphs and fit statistics. 2016 [cited 2025 Sept 12]; https://andrewpwheeler.com/2016/10/06/group-based-trajectory-models-in-stata-some-graphs-and-fit-statistics/?utm_source=chatgpt.com. Accessed 12 Sept 2025
